# BCMA in Multiple Myeloma—A Promising Key to Therapy

**DOI:** 10.3390/jcm10184088

**Published:** 2021-09-10

**Authors:** Martina Kleber, Ioannis Ntanasis-Stathopoulos, Evangelos Terpos

**Affiliations:** 1 Division of Internal Medicine, University Hospital of Zurich, 8091 Zurich, Switzerland; Martina.Kleber@usz.ch; 2 Faculty of Medicine, University of Basel, 4031 Basel, Switzerland; 3 Department of Clinical Therapeutics, School of Medicine, National and Kapodistrian University of Athens, 11528 Athens, Greece; johnntanasis@med.uoa.gr

**Keywords:** BCMA, multiple myeloma, antibody, conjugated, BiTE, ADC, CAR T cell, immunotherapy

## Abstract

Despite the discoveries of numerous agents including next generation proteasome inhibitors, immunomodulatory drugs, and monoclonal antibodies, multiple myeloma (MM) remains an incurable disease. The field of myeloma treatment in refractory or relapsed patients after standard therapy entered a new era due to the B-cell maturation antigen (BMCA) targeted approach. BCMA is a member of the tumor necrosis factor receptor family with high expression in mature B-lymphocytes and plasma cells. Given the understanding of BCMA mechanism of action in MM, BCMA plays a promising role as a therapeutic target. Several clinical trials are underway to evolve the current BCMA targeted treatment concept such as antibody-drug conjugates (ADCs), bispecific T cell engagers (BITEs) and chimeric antigen receptor (CAR) T cell therapy. Current results of representative BCMA trials may close the gap of the unmet clinical need to further improve the outcome of heavily pretreated MM patients with the potency to change the paradigm in newly diagnosed and refractory MM. This comprehensive review will give an update on various BMCA targeted treatment modalities (ADCs, BITEs, CAR T cell therapy) and its existing results on efficacy and safety from preclinical and clinical trials.

## 1. Introduction

Multiple myeloma (MM) is the second most common hematological malignancy with substantial improvements in outcome due to introduction of novel therapeutic agents such as proteasome inhibitors (PIs; bortezomib, carfilzomib, ixazomib) and immunomodulatory agents (IMiDs; thalidomide, lenalidomide, pomalidomide) [1]. The increased understanding of the pathophysiology of MM revealed in 2015 the first two immunotherapeutic agents—the monoclonal antibodies (mAbs) targeting CD38 antigen and signaling lymphocytic activation molecule family member 7 (SLAMF7) on the surface of MM cells—and enlarged standard therapeutic regimens in relapsed or refractory MM (RRMM) [2,3,4]. During the course of the MM disease, patients relapse or become refractory to PIs, IMiDs and mAbs, resulting in a very poor prognosis with a median overall survival (OS) of 5.6 months [5,6], especially in those patients with high-risk cytogenetics or failure to reach negative minimal residual disease [7,8,9]. Although new generations of IMIDs and PIs have become available, the management of these MM patients remains challenging for the clinicians [10]; hence, novel treatment modalities for RRMM disease to target current available MM therapies are indispensable.

In the therapeutic landscape of MM therapy, B-cell maturation antigen (BCMA)—one of the most specific and highly expressed antigen on myeloma cells—offers a promising target in RRMM. Evolving concepts of BCMA-targeted therapy have different mechanisms of action, including bispecific T cell engagers (BITEs), antibody-drug conjugates (ADCs) and chimeric antigen receptor T (CAR T) cell therapy [11]. The following review will highlight current insights of BCMA-targeted therapy options, updated efficacy and safety from current clinical trials, and their future clinical implication in the treatment of MM patients.

## 2. The Role of BCMA as A Therapeutic Strategy in MM

BCMA (also referred as TNFRSF17; CD269), a member of the TNF receptor superfamily 17, was initially found on the surface of normal B-lymphocytes and malignant plasma cells [12]. Thus far, two ligands of BCMA have been identified: A Proliferations-Inducing Ligand (APRIL) and B-cell activating factor (BAFF) [13], regulating B-cell maturation and differentiation in plasma cells. APRIL binds with higher affinity than BAFF for BCMA [14]. BCMA and its interaction with APRIL activate a signaling cascade via canonical and non-canonical nuclear factor kappa-B, protein kinase B (AKT) and MAPEK, resulting in proliferation, differentiation and survival of plasma cells [15,16]. Furthermore, BCMA is overexpressed in MM cells and interacts with osteoclasts in the bone marrow microenvironment, leading to their growth and the production of programmed death ligand 1 (PD-L1), and in return, osteoclasts are able to produce APRIL [2,17,18,19,20]. Membrane-bound BMCA is a substrate for the enzyme y-secretase, shedding from the surface of plasma cells, which leads to a circulated soluble BCMA form in the serum (sBCMA) [21], resulting in a decreased binding of APRIL and BAFF to membrane BCMA [21].

Elevated sBMCA levels have been identified in patients with MM and correlate with plasma cell burden in bone marrow biopsies and changes in paraprotein, potentially predicting clinical outcome [22,23]. Increased sBMCA over the median are predictive for impaired progression-free (PFS) and overall survival (OS) in MM patients; thus, patients with responsive disease after treatment showed lower sBMCA compared to MM patients with progressive MM [2]. Moreover, increased sBCMA levels in patients with monoclonal gammopathy of undetermined significance or even in smoldering myeloma patients correlate with progression in asymptomatic or symptomatic MM [24,25]. Given the short half-life (24–36 h) of sBCMA compared to IgG or IgA immunoglobulins (21 or 7 days, respectively), BCMA might be not only a biomarker for the diagnosis of MM, but also for monitoring disease progression and treatment response, even in non-secretory MM patients, for whom no accurate monitoring for therapy efficacy has been available [2]. Interestingly, previous analysis in non-secretory MM patients has shown that the bone marrow plasma cell infiltration and PET-CT scan findings—both are currently the only reliable way to monitor these patients—are associated with alterations in sBMCA levels [22,23]. Therefore, sBCMA may allow a more rapid evaluation of the disease status or efficacy of different treatment regimens compared to conventional serum M-protein [23,26]. Thus, sBCMA may allow quicker therapy decision alteration to unmasked drug resistance. These advantages are underlined by the independency of renal function and previous studies highlight that sBMCA also correlate with progression-free (PFS) and OS [26]. Sanchez et al. found that patients with higher sBCMA levels had significantly shorter PFS (3.6 months) and diminished OS (98 months) compared to patients with lower sBMCA levels (9.0 vs. 155 months, respectively) [26]. The role of membrane-bound or sBCMA levels in MM patients under anti-BCMA therapies is not clarified and further investigations are needed to analyze the correlation of baseline sBCMA and different sBCMA levels under therapy [24,27].

Currently, no approved diagnostic kit for the measurement sBCMA to diagnose or monitor MM patients is routinely available and the potential influence of anti-BCMA treatment on BCMA-expression is not quite clear; therefore, current existing conventional biomarkers in MM will still be the gold standard [23]. Although sBMCA represents a promising surrogate for prognosis and clinical response, its role as a therapeutic strategy and target is beyond doubt. With the understanding of the pathophysical mechanism of sBCMA, BMCA represents an optimal and unique target for potential therapeutic options in MM.

## 3. BMCA-Targeted Treatment in MM

Three main therapeutic types of BCMA-targeted treatment options are currently available: (1) antibody-drug conjugates (ADCs), consisting of the monoclonal antibodies (mAbs) inducing cell death through chemical links to cytotoxic agents and directed against a tumor specific antigen [28,29]; (2) bispecific antibodies (Abs) binding to BCMA-expressing B/plasma-cells and CD3ɛ-expressing T cells (BiTEs), killing tumor cells by simultaneous binding to T cell and tumor antigens; (3) anti-BCMA chimeric antigen receptor (CAR)-T cell mediated treatments (Table 1).

### 3.1. Antibody-Drug Conjugates (ADC)

The understanding of the MM plasma cells biology and its interaction within the bone marrow microenvironment build the foundation of further developments of therapeutic antigen targets in MM [30,31]. ADCs are among the fastest growing agents in plasma cell malignancies [2]. Due to their mAbs, binding ADCs to specific antigen on tumor cells, a sparing of normal cells and thus minimizing the systemic toxicity can be achieved. The main characteristics of ADCs and their toxicities are depicted in Table 2.

#### 3.1.1. Belantamab Mafodotin

Belantamab mafodotin (BM) is the first-in-class humanized afucosylated anti-BCMA IgG1 ADC, recently approved by the US Food and Drug Administration (FDA) for monotherapy in patients with relapsed or refractory MM (RRMM) [24,35,36]. Initial data in a multicenter phase I trial (DREAMM-1) in heavily pretreated (pronounced PIs and IMiDs) RRMM patients showed that BM (BMA117159) was effective with ORR of 60% (14% CR, 60% very good partial response (vgPR)) and PFS of 12 months [37] with a manageable side effects profile. Based on these data, a two-arm, randomized, phase II study of BM was conducted (DREAMM-2). The study investigated the safety and efficacy of BM in 196 MM patients refractory to IMiDs and PIs or intolerant to an anti-CD38 antibody after at least three prior therapy lines. Patients were randomized to receive either BM at the dose of 2.5 mg/kg or 3.4 mg/kg intravenous (i.v.) every 3 weeks on day 1 of each cycle until MM progression or intolerable toxicities. The ORR was 31% in the 2.5 mg/kg arm and 34% in the 3.4 mg/kg cohort, including about 20% of MM patients achieving vgPR in each therapy arm [38]. Median PFS in the 2.5 mg/kg vs. 3.4 mg/kg arm was 2.9 months vs. 4.9 months, respectively. A recently updated analysis with a median follow-up of 9 months could reach an ORR of 31% vs. 35% and median PFS of 2.8 and 3.9 months in the 2.5 mg/kg vs. 3.4 mg/kg arm, respectively [32]. Due to therapy-related adverse events (AEs) in 98% and 100%, respectively, dose modification (54% and 62%, respectively) or dose de-escalation (29% and 41%, respectively) were performed.

The most common AEs grade ≥3 were keratopathy (27% and 33%, respectively) with no difference in the median time to onset across all doses (24 days vs. 25 days, respectively). The most common symptoms of ocular toxicity presented as blurred vision and dry eye. Although the exact mechanism for the cornea AEs is unclear, a micropinocytosis-dependent internalization of ADCs in the cornea itself may cause toxicity by the linker-payload and dividing corneal epithelial cells [39,40], leading to dose reduction and/or application delays. The prophylaxis with corticosteroids eye drops showed no significant effects. Due to these data, the recommended BM dose by the FDA is 2.5 mg/kg every three weeks and ophthalmic examination at baseline and prior each BM application is required. Hematological AEs include thrombocytopenia (20% vs. 33%, respectively) and anemia (20% vs. 40%, respectively). Moreover, infusion-related events of any grade were seen in 21% in the 2.5 mg/kg dose and 16% in the 3.4 mg/kg dose. Based on these data, in 2020, the FDA granted accelerated approval to BM as the first anti-BCMA agent for RRMM patients who received at least four prior therapies with PIs, immunomodulatory agents or anti-CD38 mAB.

The potential use of ADCs combined with other treatment regimens was analyzed in a randomized phase I/II study comparing BM in the dosages of 2.5 mg/kg and 3.4 mg/kg in combination with pembrolizumab (DREAMM-4) in heavily pretreated RRMM patients. The results revealed ORR of 67% (2.5 mg/kg arm) and 14% in the 3.4 mg/kg arm [41,42]. Moreover, to encourage further combination of BM with other treatment regimens, a phase I/II trial was assessed to evaluate BM in combination with lenalidomide plus dexamethasone (arm A of DREAMM-6) or bortezomib plus dexamethasone (arm B of DREAMM-6 study) [43]. Overall response was favorable, with 78% of patients with BM (2.5 mg/kg every three weeks), and including bortezomib and dexamethasone showed that 50% of patients achieved at least a vgPR. Grade ≥3 AEs were reported in 89% of cases, with 28% of AEs leading to permanent study discontinuation of study treatment. In line with previous reported study AEs, the most relevant side effects were keratopathy and hematological side effects such as thrombocytopenia. Ongoing trials assessing the efficacy and safety of BM with different treatment combinations such as bortezomib and dexamethasone vs. daratumumab, bortezomib and dexamethasone in participants with RRMM in a randomized, open-label study (DREAMM-7 study). Additionally, the DREAMM-8 (NCT04484623) phase III study will evaluate the efficacy and safety of BM with pomalidomide and dexamethasone vs. pomalidomide plus bortezomib and dexamethasone in RRMM patients. Moreover, to analyze the efficacy of BM in combination with bortezomib, lenalidomide and dexamethasone in newly diagnosed MM patients, the DREAMM-9 study (NCT04091126) will offer new insights.

#### 3.1.2. AMG 224

AMG 224 is another anti-BCMA IgG antibody conjugated with mertansine, a derivate of maytansine and anti-tubulin inhibitor, via a non-cleavable linker [2,44]. AMG 224 demonstrated clinical activity in RRMM patients who received three or more therapy lines including PIs and IMiDs in a phase I study [33]. In the dose escalation, the maximum tolerable dose (MTD) was 190 mg applicated i.v. every 3 weeks. A total of 40 heavily pretreated RRMM patients (median of seven therapy lines) were enrolled. The results revealed an ORR of 23% with median duration of response (DOR) of 14.7 months. Comparable to BM, the most common treatment-related AEs ≥3 were hematological toxicities such as thrombocytopenia in 40%. In contrast to BM, AMG224 induced ocular events grade 1 or 2 in 30% of patients. Of note, in the AMG 224 study, no dose reduction, delays in therapy or discontinuation due to ocular AEs were performed. The AMG 224 phase I trial demonstrated a promising activity in heavily pretreated RRMM patients with a manageable toxicity profile and further clinical trials are warranted.

#### 3.1.3. MEDI2228

MEDI2228 is an ADC anti-BCMA including a fully human antibody, which provides a pyrrolobenzodiazepine (PBD) dimer conjugation via a protease-cleavable linker, leading to DNA damage and cell death. Further developments highlighted the payload tesirine as a PBD with potential anti-tumor effects and improved conjugated features [2,45]. In a preclinical model, MEDI2228 provided cytotoxic effects in myeloma and their progenitor cells independently of sBCMA levels (or p53 status) due to the strong affinity to membrane-bound BCMA than to sBCMA. The combination of MED2228 with bortezomib and DNA damage-response inhibitors have demonstrated synergistic activity [46]. Moreover, in a first-in-human phase I trial (NCT03489525), MEDI2228 was evaluated in 82 RRMM patients pretreated with PI, IMiD and mABs [34]. MM patients received 2–11 prior therapy lines and the MTD was determined at 0.14 mg/kg every 3 weeks. The most common treatment-related AEs were photophobia without keratopathy or loss of visual acuity (54%), thrombocytopenia (32%), dry eye (20%) and pleural effusion (20%). The dose of 0.14 mg/kg reached highest ORR of 61%. Overall, MEDI2228 in dose of 0.14 mg/kg every 3 weeks showed single-agent activity with a manageable side-effect profile in heavily pretreated RRMM patients.

#### 3.1.4. HDP-101

HDP-101 is new class of anti-BCMA ADC named antibody-targeted amanitin conjugates (ATAC). Amanitin is chemically synthesized from green death cap mushrooms [47] and showed a different cytotoxic payloads, due to binding to RNA polymerase II subunit A and inhibiting RNA transcription. In preclinical studies, single i.v. dose of HDP-101 showed in vitro tumor reduction and complete remission [48] with an acceptable safety profile. Based on these results, recent data by Singh et al. demonstrated efficacy of HDP-101 in cell lines with deletion of 17p [49]. These preclinical data will be the basis of further clinical investigation in MM patients in the near future.

Further investigation will provide the data of SEA-BCMA, a sugar-engineered antibody (SEA)-BCMA, defined as a naked anti-BCMA mAB without a conjugate. A phase I clinical trial (NCT03582033) is currently underway investigating the safety and effectivity of SEA-BCMA in three parts including SEA-BCMA as single agents or combined with dexamethasone and pomalidomide.

### 3.2. Anti-BCMA/CD3 Bispecific Antibodies

Bispecific antibodies (BsAbs) are molecules with affinities for two different epitopes capable of monovalent or bivalent binding on MM cells and on the CD3ɛ on T cells [50]. The strategy of therapy is that BsABs, integrating a CD3 T cell receptor binding and tumor-binding domain, are linked to tumor cells via T cells and thus create an immunological synapse, leading to a release of granzymes and perforin and inducing lysis of the target cell [51]. Moreover, the activated T cells release interferon-y and additional cytokines such as interleukin-6, -10 and tumor necrosis factor α, potentially inducing a cytokine release syndrome (CRS) with flu-like side effects such as fever, fatigue or headache in most patients [2].

Currently, there are several bispecific T cell engagers (BITEs) targeting BCMA on MM cells and CD3 receptors on T cells under investigation. Here, we present updated safety and efficacy results from early phase trials.

#### 3.2.1. AMG 420

AMG 420 (formerly BI-836909) is a BITE targeting BCMA and CD3ɛ [52]. In the first-in-human dose-escalation phase I study in 42 RRMM patients with at least two prior therapy lines including PIs and IMiDs, AMG 420 was investigated [53]. In this study, AMG 420 was given at doses ranging from 0.2 µg/d to 800 µg/d up to 10 cycles administered i.v. for 4 weeks on and 2 weeks off treatment within 6-week cycles. The ORR all over the dose escalation was 31%, but in patients (10/42) with the MTD dose of 400 µg/day, the ORR was pronounced increased with 70%. Response rates (7/10) in the 400 µg/day cohort ranked from five patients with MRD-negative complete remission (CRs), one vgPR and one partial remission (PR) with a median duration of response at the dose of 400 µg/day for 9 months. Side effects included CRS in 38%, with the majority of grade 1; only 2/42 were grade 2 and 1/42 were grade 3. Serious AEs of all grades were in 33% of infections (six of them with pulmonary infections and five with central line/catheter infections) and in 5% polyneuropathie. Although AMG 420 demonstrated high efficacy in heavily pretreated RRMM patients and toxicities were manageable, the continuous i.v. application is challenging and the focus of further developments is currently on the next-generation BCMA BITE AMG 701.

#### 3.2.2. AMG 701

AMG 701, a half-life extended form of AMG 420, is a BITE targeting the BCMA, which induces specific and effective T cell-dependent cytotoxicity against MM cells [54]. Due to a modification in the structure of the Fc fragment, the half-life could be extended up to ~112 h [2], thus offering a weekly short-term i.v. infusion. Preclinical data suggest that AMG 701 induces T cell mediated lysis of MM cells in vivo also in IMiD-resistant MM cells [55]. Given that AMG 701-mediated T cell cytotoxicity against MM cells enhances immunomodulation by IMiD pre-treatment and that the combination of IMiD and AMG 701 enhances anti-tumor effects in mouse xenograft, these results support the ongoing phase I trial, investigating AMG 701 in RRMM patients (ParadigMM-1B). AMG 701 was given as a monotherapy within three dosing categories of 5–45 µg, 0.14–1.2 mg and 1.6–12 mg i.v. weekly in 4-week cycles. Seventy-five heavily pretreated MM patients with a median of six prior therapy lines received AMG 701 [56]. Including the doses of 3–12 mg, the ORR was 36%. The most common hematological AEs were anemia (43%), neutropenia (23%) and thrombocytopenia (20%). Non-hematological AEs include with 31% diarrhea, 5% with fatigue and 25% with fever. The most pronounced AEs were CRS grade 1 and 2, which was evident in 61% of AMG 701 treated patients. These preliminary encouraging results of the ParadigMM-1B trial support further investigations of AMG 701 in MM patients.

#### 3.2.3. CC-93269

CC-93269 (formerly EM801) is a bispecific antibody binding with an anti-BCMA to BCMA and CD3 T cells. Due to its structure with an asymmetric two-arm IgG1-based humanized antibody binding bivalent to BCMA and monovalent to CD3ɛ, CC-93269 differs from other anti-BCMA T cell engagers [57]. The advantages of an extended half-life are based on an engineered Fc region enabling a weekly i.v. or s.c. application.

Initial results of a phase I trial (NCT03486067) in heavily pretreated RRMM patients (*n* = 30) with a median of five prior therapy lines and pronounced refractory to IMiDs (76%), PIs (80%) and anti-CD38 (80%) showed promising results [58]. CC-93269 was administered i.v. on day 1, 8, 15 and 22 within cycles 1–3, on days 1 and 15 within cycles 4–6 and on day 1 within cycle 7, and the doses ranged from 0.15 mg to 10 mg. The results show ORR (defined as ≥PR) of CC-93269 with 6 mg and 10 mg of 36% vs. 89%, respectively, whereas no responses were seen in patients with doses of ≤3 mg. Additionally, stringent complete response (sCR) could be achieved in 17% of patients and in the 10 mg dosing cohort, the sCR rate could be increased up to 44%. MRD negativity was achieved in 92% (12/13) of response-evaluable patients with median time to response of 4.1 weeks. Safety data concerning AEs grade ≥3 were in the majority hematological AEs with neutropenia (43%), anemia (37%) and thrombocytopenia (17%), followed by non-hematological AEs such as infections (30%). CRS of any grade was seen in 77% of patients, but pronounced with grade 1 (*n* = 15) and grade 2 (*n* = 7). In one patient with grade ≥3, who received an initial dose of 6 mg and a second dose of 10 mg, CRS was fatal. Updating safety and efficacy data of CC-93269 are awaited.

#### 3.2.4. REGN5458 and REGN5459

REGN5458 is a fully humanized antibody binding to BCMA on MM cells and to the CD3 on immune T cells, inducing T cell death [59], while cytokines release from activated T cells can be reduced. REGN5458 is currently evaluated in a first-in-human phase I/II open label study (NCT03761108) in heavily pretreated RRMM patients (PIs, IMiDs, anti-CD38) who received with 3 mg and 6 mg doses, followed by a maintenance therapy of 12 doses given every 2 weeks. Forty-five patients were enrolled and REGN5458 was escalated over six dose levels ranging from 3 mg to 96 mg. The ORR was 36% across all dose levels, whereas 60% ORR was observed in higher dose levels. Safety data for treatment-related AEs included CRS (38%, 88% for grade 1 and no patients with grade ≥3). Of note, response to therapy was durable with a DOR of ≥4 months and ≥8 months in 44% vs. 19%, respectively. Analysis of REGN5459 within a phase I trial is currently ongoing.

#### 3.2.5. PF-06863135

PF-06863135 is a fully humanized IgG CD3 bispecific monoclonal antibody that utilizes anti-BCMA and anti-CD3 targeting arms paired by hinge-mutation technology, placed in an IgG2A backbone [2,60]. Based on its structure, the half-life is 4–6 days. Limited data are available of a multicenter, open-label, phase I study (NCT03269136) in previously heavy treated RRMM patients (*n* = 17) [61], receiving PF-06863135 as a weekly non-continuous infusion i.v. within six dose-escalation cohorts. With a median of one prior therapy lines, five patients received prior BCMA-targeted treatment. Sixteen of seventeen patients could be investigated for efficacy; 6% achieved minimal response, 35% a stable disease and 53% disease progression across all dose levels. Treatment-related AEs were seen in 10/17 (59%). Most patients showed grade 1 and 2 AEs, such as CRS (24%), thrombocytopenia (24%), anemia (18%) and pyrexia (18%).

Moreover, anti-MM activity of PF-06863135 was enlarged with an s.c. dose escalation in 18 patients to reduce maximum concentration (PF-3135). The ORR was 33% and 75% with the two highest dose levels of 215 µg/kg and 360 µg/kg, respectively. The most frequent treatment-related AEs were CRS (39%), anemia (50%), thrombocytopenia (39%) and injection site reaction (33%). Of note, most of the CRS were at grade 1 and 2, and through the s.c. application, maximum concentration of PF-3135 could be reduced, allowing higher drug doses to balance side effects such as CRS. Currently, the study of PF-0601591 with or without the combination of lenalidomide is ongoing.

#### 3.2.6. Teclistamab

Teclistamab (known as JNJ-64007957 or JNJ-7957) is a Duobody characterized as a humanized IgG4 bispecific antibody binding to both BMCA and CD3 receptors of T cells. Updated results from a dose-escalation trial investigating teclistamab (NCT03145181) in previously treated RRMM patients with a PI and IMiD [62] are currently presented. Teclistamab was given i.v. (range 0.3–19.2 µg/kg (biweekly); range 19.2–720 µg/kg (weekly)) or s.c. (range 80.0–3000 µg/kg weekly) with step-up dosing used for ≥38.4 µg/kg doses. Patients received a median of five prior therapy lines with 83% triple-class refectory and 35% penta drug refractory. In the first part, no dose limiting toxicity was observed. In the safety and tolerability part of teclistamab, the most common AEs were CRS (70%; grade 3/4 0) and neutropenia (60%; ≥grade 3 40%); neurotoxicity grade 1 was reported in one (3%) patient. Of note, the median time to CRS onset was longer with s.c. vs. i.v. application. The ORR in response-evaluable patients treated within the second part (*n* = 40) was 65%, whereas 58% achieved ≥vgPR (30% ≥CR) with median duration of response not reached. Teclistamab showed encouraging efficacy (weekly 1500 µg/kg s.c.), with deepening responses supporting further investigation as monotherapy or with other drug combinations.

#### 3.2.7. TNB-383B

TNB-383B differs from other BCMA/CD3 bispecific antibodies due to its characteristic with one arm with heavy and light chain with αCD3 and a second arm only with heavy chain, binding dually with high-affinity to BCMA domains [63]. Due to its modification, αCD3 binds and activates CD4/CD8 T cells preferentially; thus, release of cytokines could be reduced, potentially leading to a reduced incidence of CRS [64]. Initial results of a first-in-human phase I/II dose escalation study (NCT03933735) in RRMM patients with a minimum of three prior therapy lines of PIs, IMiDs and anti-CD 38 mAb are currently presented [65]. TNB-383B was given in a dose escalation of 0.025 to 50 mg i.v. every 3 weeks in patients with RRMM who received median of seven therapy lines. Initial safety results of 38 patients revealed as the most common AEs CRS grade 1 or 2 (21%) and headache (13%). Preliminary ORR of 52% at doses ≥5.4 mg with a durable response (~24 weeks) in a 3-week dosing was observed.

### 3.3. Anti-BCMA CAR-T Cells in MM

CAR strategy targeting BCMA is innovative in cellular immunotherapy with the characteristics of combining target specificity of mAbs and the cytotoxicity of T cells. The main advantage of CAR-T cells is that they, in contrast to human leukocytes antigen (HLA)-restricted T cell receptors, are not HLA-restricted, leading to an HLA type independent therapy strategy [66]. Multiple clinical trials have shown promising therapeutic efficacy of CAR-T cells in patients with relapsed/refractory B cell neoplasms [67,68,69,70]. Processes include CAR-T cells being produced after leukapheresis of peripheral white blood cells of the patients or healthy donors to obtain CD3+ T cells for autologous vs. allogeneic CAR-T cells. Thereafter, the collected white blood cells, following a stimulation of T cells, express CD3 and CD28 or 4-1-BB by coated beads with mAb [39]. In a subsequent step, these activated T cells are transduced with a gene via a lentiviral vector, which encodes a receptor to the tumor-specific antigen on tumor cells, and are able to express CAR gens [39]. The main characteristic of CAR-T cells is that they use a CAR against antigen of tumor cells such as BCMA, CD19, CD138 (syndecan-1), Orphan G-protein-coupled receptor class C group 5 member D (GPRC5D) and immunoglobulin kappa light chain [71,72,73]. The role of BCMA in the pathogenesis of plasma cell malignancies offers a novel and attractive strategy for CAR-T cells [74]. Table 3 shows the main characteristics and differences between first and second generation CAR T cell constructs.

#### 3.3.1. Autologous BCMA CAR-T Cells

##### Idecabtagene Vicleucel (bb2121)

Idecabtagene Vicleucel (former Ide-cel; bb2121) is a second generation BCMA targeted CAR-T cell incorporating a single chain variable fragment, a 4-1BB co-stimulating domain and a CD3Zeta signaling domain, inducing cytotoxicity against MM cells independently of BCMA expression or soluble BCMA [39,75]. Registrational indications of idecabtagene vicleucel CAR-T therapies include single-arm studies with RRMM patients who were initially triple-class-exposed or triple-class-refractory [27]. In a multicenter phase I trial (CRB-401), the efficacy of bb2121 in RRMM patients who received more than three prior therapy lines (including PIs and IMiDs) showed promising results [75,79]. The ORR was overall 85% (CR 45%, sCR 36%), with a median PFS of 11.8 months (95% CI 6.2 to 17.8). The doses of bb2121 ranged from 150 × 10^6^ to 800 × 10^6^ CAR-cells. Treatment-related AEs included hematological toxicity such as neutropenia (92%), leukopenia (61%), anemia (58%) and thrombocytopenia in 58% [75]. CRS was evident in 76% of all patients (≥3 grade 6%). A follow up study phase II of bb2121 [80] was presented in the KarMMa study on 128 patients who received 150 × 10^6^–450 × 10^6^ CAR-T cells after 3 days of lymphodepletion (cyclophosphamide 300 mg/m^2^ + fludarabine 30 mg/m^2^) and with prior therapy lines including PIs, IMiDs and anti-CD38 antibodies [80]. Within the primary endpoint, ORR was 73% and secondary endpoints included CR rate, duration of response and MRD. CR/sCR in all doses was 33%, with a median time to response of one month and a duration of response of 10.7 months. Patients achieving a negative MRD within at least a vgPR was evident in 39% and 26% in patients within a CR. The median PFS in patients with a bb2121 in the dose of 450 × 10^6^ was 8.6 months (overall, 19.4 months). The main toxicities included CRS in 84% (grade 3 with 5% and one patient each with grade 4 and 5). Additional AEs were manageable with cytopenia and reversible neurotoxicity.

##### LCAR-B38M/JNJ-4528

LCAR-B38M/JNJ-4528 is a compound dual epitope targeting CAR-T cells that binds two BCMA epitopes, thereby differing from other CAR-T cells, which is currently investigated in the a single-arm, open-label, multicenter phase I study in RRMM patients (LEGEND-2 study) (NCT03090659). The LEGEND-2 study enrolled 57 RRMM patients showing an ORR of 88% and MRD negativity in 63%, after a median follow-up of 8 months [81]. The most common AEs were pyrexia in 91%, CRS (90%) and hematological AEs such as thrombocytopenia in 49% and leukopenia in 46%. Updated results from the LEGEND-2 trial with a long-term follow-up of 25 months [82] showed an enlarged PFS of more than two years in patients with a CR response and a median duration of response of 29.1 months.

Ciltacabtagene autolecel (cilta-cel, known as LCAR-B38M/JNJ-4528) was further investigated in the CARTITUDE-1 trial, a phase Ib/II study. Updated results have been presented [76] and show that cilta-cel was associated with deep (ORR 100%, sCR in 86%) and continued response in RRMM patients within the median follow-up of 11.5 months. The 9-month PFS rate was 86% (95% CI, 67–95). Safety results including grade 3–4 hematological AEs with neutropenia, anemia and thrombocytopenia were present in 91%, 68% and 60% of included patients, respectively.

There are ongoing studies including the investigation of LCAR-B38M/JNJ-4528 in phase II and III trials: (1) CARIFAN-1 trial, a phase II study investigating the efficacy and safety of LCAR-B38M/JNJ-4528 CAR-T cells in RRMM patients (NCT03758417) with primary outcome of ORR; (2) CARTITUDE-2 trial, evaluating the efficacy and safety of JNJ-4528 (NCT04133636) with a focus on analyzing the MRD-negative rate in patients receiving a single infusion of LCAR-B38M/JNJ-4528 with or without lenalidomide; and (3) CARTITUDE-4, a phase III study analyzing the efficacy of CAR-T cells in patients with one or three prior therapy lines compared to standard-of-care treatments such as pomalidomide, bortzomib and dexamethasone (PVd) or daratumumab, pomalidomide and dexamethasone (DPd) in MM patients relapsed and lenalidomide refractory (NCT04181827).

##### Orvacabtagene Autoleucel (JCARH125)

Orvacabtagene autoleucel (orva-cel) is a BCMA-directed CAR-T cell with a fully human binder, which has shown in vitro activity, even in patients with low-BCMA cells [77]. Due to its optimized architecture, JCARH125 decreases antigen-independent collapse by improving BCMA binding on target cells, and the addition of purified CD4+ and CD8+ CAR-T cells with memory phenotype with high durability [24]. There are up-to-date results of the phase I/II trial (EVOLVE trial) of 51 patients treated with orva-cel at 300, 450 and 600 × 10^6^ CAR-T cells after lymphodepletion with fludarabine and cyclophosphamide [83]. Patients with a median age of 61 years (range: 33–77 years) and a median of six prior therapy lines were included. The efficacy results revealed an ORR of 91% with CR/sCR rate of 39%. Two patients showed dose-limiting toxicities with a grade 3 neurological event at a dose of 300 × 10^6^ CAR-T cells and grade 4 neutropenia at 450 × 10^6^ CAR-T cells. The most common hematological AEs were grade 3 anemia, neutropenia and thrombocytopenia in 21%, 55% and 44% of patients, respectively. Grade 3 or higher infections occurred in 14% of patients. CRS were manageable (CRS grade ≥ 3: 2%) with tocilizumab with or without steroids in 78%, anakinra in 14% and/or vasopressors in 6%. Overall, orva-cel at the doses of 300, 450 and 600 × 10^6^ CAR-T cells demonstrated a compelling efficacy in heavily pretreated pts with RRMM.

Technical advances of CAR-T cells manufacturing in MM continue to evolve in terms of disease relapse and toxicity. To address these topics, CARs such as cilta-cel (known as LCAR-B38M/JNJ-4528) targeting double epitopes of BCMA have been developed. With the latter, cilta-cel compared to ide-cel and orva-cel could increase its binding affinity even in MM cells with low BCMA expression, resulting in lower CAR-T cell doses and in a delay of the median time of onset of CRS, with 1–2 days vs. 7 days (ide-cel vs. cilta-cel). These effects allow the management of cilta-cel infusion in outpatients following hospital admission after 5 days to monitor potential CRS [84,85].

##### Clinical Development of CAR-T cell and BCMA Targeted Treatment

The improvement of the efficacy of CAR-T cells in MM will be the focus for the development of the next generation of CAR-T cells. The main characterization will be in producing a multi-specific CAR-T cell responding to lower levels of targeted-antigen on MM cells and to improve the anti-tumor immune response [39].

##### Bispecific CAR-T

Eligibility for therapy with BCMA CAR-T cells could be limited due to the inconsistent expression of BCMA in MM cells [86]. Zah et al. developed systemically optimized single-chain bispecific (OR-gate) CARs that target BCMA and CS1 [87]. It was demonstrated that 90% of MM samples expressed CS1 [88]. BCMA/CS1 OR gate CAR-T cells are able to eliminate heterogeneous MM cells in vitro and in vivo, offering a promising therapy option in the landscape of CAR-T cell therapy against myeloma cells [24].

##### CT103A

CT103A is a novel second generation CAR, which incorporates a fully human scFv sequence. Compared to the second generation CAR-T cells of ide-cel and cilta-cel, the novelty of CT103A is the incorporation of a fully human scFv sequence, potentially increasing its clinical efficacy compared to CARs including a non-human originated antigen-targeting domain. Initial results of a single-center study (ChiCTR1800018137) including 16 RRMM patients with three prior therapy lines (PIs and IMiDs) showed an ORR of 100% in six patients with CR/sCR and two patients with vgPR [78]. MRD negativity could be achieved in 15/16 patients. CRS was present in all patients—10 patients with grade 1–2, 5 patients with grade 3 and 1 patients with grade 4. One patient died due to lung infection 19 days after application. In the peripheral blood, CT103A was highest at day 14 and was still detectable in 12 patients. The results highlight the fast expansion and persistence of CT103. Due to these results, CT103 offers a promising approach in patients with RRMM.

##### Decartes-08

Decartes-08 is an RNA-generated anti-BCMA CD8 CAR-T cell, which effectively depleted MM cell lines and MM cells from patients with NDMM and RRMM through production of cytokines such as IFNµ, TNFα and IL-2 [89]. The grade of cytokines response correlate with the expression of anti-BCMA CAR expression with the advantage of reduced risk of CRS in comparison to virus-generated CARs [90]. Decartes-08 also showed efficacy in high-risk MM patients with plasma cell leukemia (NCT03448978).

#### 3.3.2. Allogeneic BCMA CAR-T cells

An alternative therapeutic approach offers BCMA 1-R2 CAR-T cells, which are anti-BCMA allogeneic CAR-T cells from healthy donors [91]. To reduce the risk of graft versus host disease (GvHD), TRAC gene was replaced by transcription activator-like effector nucleases (TALEN). Moreover, CD52 was additionally knocked out by TALEN to make BCMA 1-R2 CAR-T cells resistant to anti-CD52 antibodies (e.g., alemtuzumab). The purpose of manufacturing CD52-deficient T cells is to allow the application of alemtuzumab concurrently or prior to engineered T cells, which potentially eliminate CD52 expressing host T cells, to mediate engraftment and avoid allorejection [92,93]. Moreover, the safety of BCMA 1-R2 CAR-T cells is enhanced by the incorporation of intra-CAR rituximab binding domain as an off switch, enabling the elimination of CAR-T with rituximab [94].

## 4. Conclusions

Despite the enlarged armamentarium of different therapy options (e.g., PIs, IMiDs, and monoclonal antibodies) in recent years, MM is still an incurable disease that needs continuous therapy improvements. The discovery of BCMA offers a new era in the field of immunotherapy in anti-myeloma therapy, including ADC, anti-BCMA/CD3 bispecific antibodies or anti-BCMA CAR-T cell therapy. Early clinical trials of BCMA-targeted immunotherapies offered promising results in efficacy and tolerable side effects in RRMM patients with several prior therapy lines. Therapy decision-making in patients with MM should be based on patient-related factors (e.g., patients’ preference, comorbidities) and disease-related risk factors (biological MM features, numbers and type of prior therapies, access to novel agents) to balance effectivity and toxicities. Based on these considerations, an important matter is the decision for appropriate anti-BCMA immunotherapy options.

It is not clear which anti-BCMA approach is superior to the others; therefore, the decision for a suitable anti-BCMA therapy for each individual MM patient will be the challenge [95]. The ADC therapy is an attractive and easy applicable therapy, but it bears the limitation of the corneal toxicities and needs further understanding to optimize its management. The bispecific antibodies are off-the-self therapies without need for ex vivo manipulation of MM cells and have potential for direct use. The main disadvantage of bispecific antibodies is their short lifetime, which needs prolonged i.v. infusion time via central venous access. Amid its ongoing investigation in clinical trials, the focus is on the improvement of bispecific antibodies with prolonged half-lives. CAR-T cells offer an attractive therapy option, due to promising results with deep response rates similar to frontline therapy in an MM patient population with heavy prior therapy lines. Moreover, the main advantage is HLA-independency with universal application. The main disadvantages of CAR-T cells include manufacturing expense, bridging with chemotherapy and need for several hospital stays in specialized centers with appropriate infrastructure; the side effects including CRS, neurotoxicity and limited therapy effects after one year. A “head to head” comparison of anti-BCMA targeted therapy will be difficult, but ongoing trials facing optimized response with manageable toxicity and the incorporation in earlier therapy lines will further improve the landscape of MM therapy.

Therefore, further investigations will focus on anti-BCMA based therapy alone or in combination with other anti-myeloma drugs as an evolving concept in patients with relapsed or refractory MM or even in newly diagnosed MM to consistently improve the outcome towards a cure.

## Figures and Tables

**Table 1:** Main differences of various BCMA targeted therapies in multiple myeloma.

	Antibody-Drug Conjugates (ADSs)	Bispecific T cell Engagers (BITEs)	CAR-T Cell Therapy (CAR-T cells)
**Drug**	Belantamab mafodotin	AMG 420	Idecabtagene Vicleucel
	AMG 224	AMG 701	LCAR-B38M/JNJ-4528
	MEDI2228	CC-93269	Orvacabtageneautoleucel
	HDP-101	REGN5458	CT103A
		PF-06863135	Decartes-08
		Teclistamab	
		TNB-8383B	
**Characteristics**	Consisting of the monoclonal antibodies inducing cell death through chemical links to cytotoxic agents and directed against a tumor specific antigen	Binding to BCMA-expressing B/plasma-cells and CD3ɛ-expressing T cells (BiTEs), the latter killing tumor cells by simultaneous binding to T cell and tumor antigens.	Consisting of tumor-associated antigen-targeted single chain variable fragment connected with intracellular and co-stimulating domains
**Logistics**	Off-the-shelf	Off-the-shelf	Sustained manufacturing time
**Application**	Intravenous	Continuous intravenous infusion; short-life time	Manufacturing expense, bridging with chemotherapy with need of several hospital stays
**Duration of Therapy**	Every three weeks	Mostly weekly until progression	One time therapy
**Main Adverse Effects**	Corneal toxicities	CRS	CRS
	Hematological toxicities	Hematological toxicities	Neurotoxicity
	Infusion-related side-effects		Hematological toxicities

BCMA: B-cell maturation antigen; CRS: cytokine release syndrome; BiTEs: bispecific T cell engagers; CAR: chimeric antigen receptor; ADCs: antibody-drug conjugates.

**Table 2:** Main characteristics of different ADCs and their toxicities in multiple myeloma.

Agent	Effector Moiety	Structure	Mechanism of Action	Main Toxicity Profile
Belantamab mafodotin	Monomethyl auristatin F	humanized IgG1 Ab conjugated to a microtubule disrupting agent MMAF	tubulin inhibitor	thrombocytopenia, corneal events [32]
AMG 224	Maytansinoid DM1	comprised of an anti-tubulin inhibitor maytansine derivative conjugated to antibody lysine residues via the non-cleavable 4-(N-maleimidomethyl) cyclohexane-1-carboxylate linker	ubulin inhibitor	Thrombocytopenia grade ≥ 3: 40% ocular AEs grade 1–2: 30% [33]
MEDI2228	Pyrrolo-benzodiazepine	fully human anti-BCMA antibody site-specifically conjugated to a PBD tesirine via a protease-cleavable linker	DNA damage	photophobia without keratopathie or loss of visual acuity (54%), dry eye (20%) thrombocytopenia (32%), pleural effusion (20%) [34]
HDP-101	Amanitin	fully humanized mAb conjugated to amanitin via a non-cleavable MC linker	RNA polymerase II inhibitor	preclinical

Abbreviation: ADC antibody-drug conjugates; DM1: *N*^2′^-deacetyl-*N*^2′^-(3-mercapto-1-oxopropyl)-maytansine; (m)Ab (monoclonal) antibody; MMAF monomethyl auristatin; PBD pyrrolobenzodiazepine.

**Table 3:** Characteristics of selected first and second generation CAR-T cells.

CAR-T generation	Antigen Binding Domain	Construct	Transduction	Toxicities
First generation				
Containing only a signaling domain [74]		CD3ζ signaling domain		
Second generation				
Incorporatinga co-stimulatory domain (4-1BB, CD28, and/or OX-40)				
Idecabtagene Vicleucel (former Ide-cel; bb2121) [75]	murine scFv	4-1BB costimulatory domain, CD8α hinge and transmembrane domains, culture/activation medium: anti-CD3and anti-CD28, OKT3	lentiviral vector	CRS (all grade): 76% CRS grade ≥ 3: 6% median time to CRS onset: 2 days, median CRS duration: 5 days, neurotoxicity: 42% (including 1 grade 4)
Cilta-cel (LCAR-B38M/JNJ-4528) [76]	bispecific variable fragments of heavy-chain antibodies; targeting 2 distinct BCMA epitopes	CD3ζ and 4-1BB	lentiviral vector	Most common AEs: pyrexia (91%), CRS (90%), median time to CRS onset: 9 days, median duration of CRS: 9 days, thrombocytopenia (49%), leukopenia (46%)
Orvacabtagene autoleucel (JCARH125) [77]	human scFv	optimized spacer, CD28 transmembrane domain, optimized spacer, 4-1BBcostimulatory domain	lentiviral vector	CRS grade ≥ 3: 2%, neurologic events grade ≥ 3: 7% of pts
CT103A [78]	human scFv	CD8α hinge and transmembrane region, 4-1BB costimulatory domain	lentiviral vector	CRS in all patients: 10 pts grade 1–2, 5 pts with grade 3 and 1 pts with grade 4

Abbreviation: AE adverse event; BCMA B-cell maturation antigen; CAR chimeric antigen receptor; CRS cytokine release syndrome; DLT dose-limiting toxicity; scFv single-chain variable fragment; pts: patients.

## Data Availability

Data are available upon request from the corresponding author.
